# Clinician perspectives of the implementation of an early intervention service for eating disorders in England: a mixed method study

**DOI:** 10.1186/s40337-024-01000-4

**Published:** 2024-04-05

**Authors:** Katie L Richards, Matthew Phillips, Luiza Grycuk, Lucy Hyam, Karina Allen, Ulrike Schmidt

**Affiliations:** 1 Centre for Research in Eating and Weight Disorders, Department of Psychological Medicine, Institute of Psychiatry, Psychology and Neuroscience, Kings College London, De Crespigny Park, London, SE5 8AB UK; 2 Centre for Implementation Science, Health Service and Population Research Department, Institute of Psychiatry, Psychology and Neuroscience, King’s College London, De Crespigny Park, London, SE5 8AF UK; 3 Eating Disorders Outpatient Service, South London and Maudsley NHS Foundation Trust, Denmark Hill, London, SE5 8AZ UK

**Keywords:** Early intervention, Eating disorders, Normalisation process theory, Implementation

## Abstract

**Background:**

The First Episode Rapid Early Intervention for Eating Disorders (FREED) service has been shown to reduce the wait for care and improve clinical outcomes in initial evaluations. These findings led to the national scaling of FREED in England. To support this scaling, we conducted a mixed method evaluation of the perceptions and experiences of clinicians in the early phases of scaling. The Normalisation Process Theory (NPT) was used as a conceptual lens to understand if and how FREED becomes embedded in routine practice.

**Methods:**

The convergent mixed method evaluation included 21 semi-structured interviews with clinicians from early adopter sites and 211 surveys administered to clinicians before, immediately after and 3 months after the FREED training. The interview guide and survey included questions evaluating attitudes towards early intervention for eating disorders (EDs) and NPT mechanisms. Interview data were analysed using an inductive thematic analysis. The NPT was applied to the inductively derived themes to evaluate if and how NPT domains impacted the implementation. Survey data were analysed using multilevel growth models.

**Results:**

Six themes and 15 subthemes captured barriers and facilitators to implementation at the patient, clinician, service, intervention, implementation and wider system levels. These interacted with the NPT mechanisms to facilitate or hinder the embedding of FREED. Overall, clinicians were enthusiastic and positive towards early intervention for EDs and FREED, largely because of the expectation of improved patient outcomes. This was a considerable driver in the uptake and implementation of FREED. Clinicians also had reservations about capacity and the potential impact on other patients, which, at times, was a barrier for its use. The FREED training led to significant improvements in positive attitudes and NPT mechanisms that were largely maintained at the 3-month follow-up. However, negative attitudes did not significantly improve following training.

**Conclusions:**

Positive attitudes towards early intervention for EDs increased enthusiasm and engagement with the model. Features of the model and its implementation were effective at developing adopter commitment and capabilities. However, there were aspects of the model and its implementation which require attention in the future (e.g., capacity and the potential impact on the wider service).

**Supplementary Information:**

The online version contains supplementary material available at 10.1186/s40337-024-01000-4.

## Abstract (plain language summary)

First Episode Rapid Early Intervention for Eating Disorders (FREED) is an early intervention service for 16-to-25-year-olds who have had an eating disorder (ED) for three years or less. Studies have shown that FREED can reduce the length of time that people wait for treatment and that it improves outcomes. However, it was unclear what it was like for clinicians trying to deliver the service. We used questionnaires and interviews with clinicians who were preparing to or already delivering FREED to understand what they thought about it and their experiences of delivering it. Overall, clinicians were positive and enthusiastic about early intervention for EDs and FREED because they believed that it could improve patient outcomes. This enthusiasm and features of the FREED model and support facilitated its delivery. However, clinicians were worried about their ability to deliver FREED with limited resources and the impact this might have on patients who are not eligible for the service. Resources were a major barrier to providing treatment as quickly as possible. This study suggests that FREED was well-received by those delivering it and that many parts of the service and support were helpful, but resource issues need to be addressed in the future.

**Supplementary Information:**

The online version contains supplementary material available at 10.1186/s40337-024-01000-4.

Eating disorders (EDs) are disabling mental health conditions characterised by mentally and physically damaging eating and weight-control behaviours. Eating, weight, and shape concerns can dominate thinking and substantially interfere with the person’s life [1]. While there has been progress in the treatment of EDs, post treatment remission rates remain modest with ∼20–30% of individuals continuing to be ill 10 years post onset [2, 3]. Providing evidence-based treatments as early as possible following illness onset, when symptoms are potentially more amenable to change, could further improve the effectiveness of treatments [4]. This is particularly pertinent given the 2-5-year period between the onset of EDs and their treatment [5].

Evaluations of the First Episode Rapid Early Intervention for EDs (FREED) service suggest that providing treatment early may improve treatment outcomes [6]. FREED is an early intervention service for 16-to-25-year-olds (‘emerging adults’) who have had an ED for 3 years or less [7]. The FREED service model has been described in detail elsewhere [8, 9]. In brief, a group of clinicians embedded within an existing ED service allocate some of their time to deliver FREED, including a FREED Champion who oversees the service. Key features of the service model include a 48-hour engagement call, wait time targets of 2-weeks for assessment and 4-weeks for treatment, and a care package, which adapts treatment to the needs of emerging adults in early-stage illness [8]. Compared to treatment as usual, FREED has been shown to significantly reduce the wait for treatment, duration of untreated ED (DUED) and improve treatment uptake and outcomes [6, 10–12]. These initial positive findings led to additional funding to scale FREED nationally in the National Health Service (NHS) in England [8]. The national scaling was collaboratively led by the South London and Maudsley (SLaM) NHS Foundation Trust, King’s College London (KCL) and the Academic Health Science Network (AHSN) [13].

Successfully scaling an intervention beyond its originating centre is widely recognised as challenging, complex, and unpredictable [14, 15]. The field of implementation science and research has developed rapidly over the past 20 years with the aim of untangling this complexity and identifying evidence-based approaches to increase successful implementation and scaling. The implementation approach for FREED was developed with reference to a widely used implementation science framework, namely the RE-AIM framework [8]. The RE-AIM framework outlines five dimensions (Reach, Effectiveness, Adoption, Implementation, and Maintenance) that dynamically interact to determine the broad and equitable population-based impact of a new evidence-based intervention, policy or programme [16, 17]. Key implementation strategies used during the national scaling of FREED included educational materials, a training package, implementation support, and a FREED Network with quarterly data collection and feedback (see Additional file 1 for more details). The FREED Network included all sites that were preparing to or implementing FREED and aimed to facilitate communication and collaboration across these services [8]. There have been limited evaluations of the implementation of early intervention in EDs and FREED [9, 18]. Richards et al. [9] evaluated fidelity to the FREED wait time targets and care package during the initial scaling evaluation, and Hyam et al. [18] investigated the views of the AHSN implementation programme leads during the rapid national scaling.

Another important factor to evaluate early in any implementation or scaling endeavour are the attitudes and perceptions of clinicians delivering the intervention, particularly the perceived value and feasibility of the intervention [15, 19]. During the preparation of this study, we found only one study that evaluated clinician attitudes towards early intervention for EDs. Specifically, a national survey in Italy found that 71% of respondents considered early intervention for EDs to be “very important”, second only to psychosis [20]. However, it has long been known that while positive attitudes and intentions are important, they do not always result in actions or behaviours [19]. Social and contextual obstacles, such as capacity, infrastructure, and a lack of management support, have been shown to hinder even the most enthusiastic clinician [21]. It is therefore essential that we look beyond attitudes and consider other factors that may impact the implementation and embedding of FREED. The Normalisation Process Theory (NPT), another widely used theory within implementation science, has been shown to facilitate evaluations of the implementation and embedding of health interventions in routine practice [22]. NPT focuses on the individual and collective ‘work’ that individuals delivering the intervention must engage in to implement and embed a new practice. It operationalises this work through four generative mechanisms: [1] *coherence*: the sense-making work that people do individually and collectively when trying to define, operationalise, and understand the meaning, uses and utility of an intervention; [2] *cognitive participation*: the relational work that people do to create and sustain engagement and a community of practice around an intervention; [3] *collective action*: the mental and material work that people do to enact a set of practices, includes individual and collective purposive action, allocation of resources and training, building confidence/accountability and reshaping and reorganising behaviours and contexts; and [4] *reflexive monitoring*: the individual and collective appraisal work that people do to assess the impact and value of an intervention [22–25]. The NPT was developed iteratively over nine years using empirical generalisations from implementation studies, formal theory-building approaches and “road-testing” [26]. A growing body of literature demonstrates the value of the NPT in planning and evaluating implementation [27, 28]. Given NPT’s demonstrated utility and the analytical level of this study (focused on the views and experiences of clinicians), it was chosen as the conceptual lens for this study to aid our analysis and understanding of how FREED is implemented and embedded.

To support the scaling of FREED, we conducted a mixed methods evaluation of clinician attitudes towards and experiences of implementing FREED. The overarching aim was to understand clinician attitudes towards early intervention for EDs and FREED, implementation processes and barriers and facilitators to implementation. Qualitative and quantitative data were collected in parallel to address this aim. Qualitative interviews with early adopters of FREED were used to provide an in-depth understanding of the attitudes towards early intervention and FREED and perceived barriers and facilitators. The NPT domains informed the interview questions and were then applied to the data to further explore if and how FREED may be implemented and embedded in routine settings. The quantitative data were collected to complement these qualitative interviews. Specifically, quantitative surveys were administered to a wider variety of FREED services and clinicians to gain a broader understanding of the degree to which clinicians hold positive or negative attitudes towards early intervention for EDs and the level of NPT ‘work’ taking place. The quantitative survey was also used to evaluate the impact of one of the key hypothesised facilitators for FREED, namely the FREED training. The FREED training was hypothesised to facilitate the implementation of FREED through improved knowledge and attitudes towards early intervention and increased NPT ‘work’. A mixed methods approach was used to provide a comprehensive picture of attitudes towards early intervention for EDs and FREED, the role of NPT implementation processes and barriers and facilitators to implementation. The findings of this study can be used to further shape and refine the FREED model and its implementation and may be of great interest to anyone considering or already developing and/or implementing early intervention for EDs. The study highlights key factors that can facilitate the translation and spread of ED research into practice.

## Methods

### Study design and procedure

This study was a convergent mixed method evaluation of clinician attitudes and experiences of early intervention for EDs and FREED [29]. This study was conducted between September 2019 and April 2021, shortly after the multi-site FREED-Upscaled study finished [11] and early in the national scaling process. During this time, the number of ED services implementing FREED grew from five (in September 2019) to 22 (in April 2021), most of which began implementing FREED from December 2020 onwards.

For the qualitative component, clinicians working in eight urban and rural FREED services in England were purposively invited by email to complete a semi-structured interview. The FREED Champion at each site were invited to take part and asked to invite members of their team with a range of experiences with FREED and EDs. Due to the recruitment approach, the total number of people invited to take part at each site is unknown. All interviewed services were specialised ED services and early adopters of FREED (i.e., planned to or began implementing FREED before rapid national scaling). Seven were adult ED services and one was a 0-to-25-year-old ED service. The eight sites were selected because they were early adopters of FREED and had been implementing the model for at least 5 months at the time of interview (range: 5 to 72 months). A topic guide (see Additional file 2) was flexibly used to guide the interviews. The topic guide was structured according to the four NPT domains and included questions on attitudes towards, and experiences of, early intervention for EDs and FREED. All interviews were conducted by KR (see Additional file 3 for researcher description). The average length of the interviews was 63 min (range = 32 to 118 min), and they were conducted over the phone (*n* = 15), in-person (*n* = 2) or via video calls (*n* = 4). The interviews were recorded and transcribed verbatim. KR’s role, prior relationship to participants, and emerging ideas were carefully considered through reflective note taking. Potential reporting biases were addressed by stressing the importance of understanding positive and negative views/experiences and the confidentiality of the data collection.

For the quantitative component, clinicians attending the 1-day in-person/virtual FREED training were invited to participate by email before attending the training. For those that agreed to participate, a questionnaire pack was administered before, immediately after, and three months after the training. The questionnaire was intended to collect information on attitudes towards early intervention for EDs and the NPT ‘work’ taking place and the impact of the training on these. The questionnaire pack consisted of demographics, an ‘attitudes towards early intervention for EDs’, and the Normalization MeAsure Development (NoMAD) questionnaires. Questionnaire participants were from 31 different ED services across England, most of which were preparing to or recently launched FREED.

### Questionnaires

#### Attitudes towards early intervention for EDs questionnaire (see additional file 4 for questionnaire development):

a 15-item self-reported attitudes questionnaire was developed for the study. The questionnaire includes items measuring positive attitudes, negative attitudes and the importance of early intervention for EDs. Items measuring beliefs about knowledge and skills to implement early intervention were also included. Most items were rated on 7-point Likert scales ranging from 1 = “strongly disagree” to 7 = “strongly agree”. Items measuring the importance of early intervention were rated on 5-point Likert scales ranging from 1 = “not important” to 5 = “absolutely essential”. The Cronbach’s alpha for the subscales were α = 0.72 (positive attitudes), α = 0.72 (negative attitudes), α = 0.57 (knowledge/skills), and α = 0.67 (importance of early intervention for EDs).

#### Normalization MeAsure Development (NoMAD; 30):

a 23-item self-report questionnaire measuring the four NPT constructs. The global normalisation items were not included in this analysis. Items were rated on 5-point Likert scales ranging from 1 = “strongly disagree” to 5 = “strongly agree”. If an item was not relevant, participants could also select ‘not relevant to my role’, ‘not relevant at this stage’, and ‘not relevant to the intervention’. Internal consistencies for each subscale were α = 0.56 (coherence), α = 0.76 (cognitive participation), α = 0.72 (collective action), and α = 0.67 (reflexive monitoring).

### Analysis

The qualitative analysis was conducted by KR using NVivo 12 [31]. A critical realist perspective was adopted for the qualitative analysis [32]. The qualitative analysis was completed in two stages [33]. In *stage 1*, an inductive reflexive thematic analysis was conducted according to the framework outlined by Braun and Clarke [34, 35]. Segments of each transcript relevant to the study aim were coded. Codes were then grouped and organised into themes based on recurring experiences. In *stage 2*, each NPT mechanism was applied to each subtheme using standardised definitions of each NPT construct (outlined in the introduction) and interrogating questions to establish if and how NPT ‘work’ contributed to each subtheme. The results of this application were qualitatively summarised in a table.

Given the epistemic approach, data saturation was not used to determine sample size [36]. Instead, the aim was to interview at least two clinicians per team. However, this was not possible for two sites due to COVID-19 and capacity issues. The trustworthiness and credibility of the results were evaluated by distributing the findings of the inductive thematic analysis to four participants for comments and feedback. All participants felt that the results were an accurate reflection of their experiences. While intercoder reliability (ICR) is not typically used as a measure of quality in reflexive thematic analysis [37], a portion (20%) of the interviews were coded by an independent researcher (MP). The aim of this analysis was not to control for or entirely remove researcher subjectivity from the analytical process, but to evaluate whether the researcher’s role and biases impacted the analysis to such an extent that similar themes would not be identified by an independent researcher. The percentage of agreement between the coders (KR and MP) was high (> 90%) across all codes and 90% of codes obtained moderate to almost perfect Cohen’s kappa values. Differences in coding were discussed and resolved.

The quantitative analysis was conducted after the qualitative analysis in SPSS version 27 [38] and R [39]. Means, standard deviations and percentage disagreement/agreement were calculated for attitude and NoMAD questionnaire items and subscales at each time point. Multi-level models (MLM) were used to evaluate the impact of the FREED training on NoMAD and attitude subscale scores over time. MLMs were fit using the steps outlined by Bliese [40] and using the ‘nlme’ package in R [41]. In all models, the participant random intercepts were nested within training session. Linearity, homogeneity of variance, and normality assumptions were visually examined. All models were fit with a quadratic time function to account for the non-linear slope. Where applicable, heterogeneity of variance was accounted for in the models’ error structure. All clinicians who attended the FREED training between September 2019-April 2021 were invited to participate in the survey (*n* = 296). 75% (*n* = 211) completed at least one questionnaire. Of these, ∼86% (*n* = 185), ∼72% (*n* = 154), and ∼54% (*n* = 115) completed questionnaires at time 1, 2 and 3, respectively. Reasons for missing data or attrition were unknown. Baseline demographics did not predict missingness.

The results of the qualitative and quantitative findings were merged by matching the questionnaire items to the qualitative data and comparing the similarities and dissimilarities in the results. The qualitative and quantitative findings are presented alongside each other in two sections in the results. In the first section, we summarise the findings of the inductive reflexive thematic analysis (*stage 1*) alongside responses to the attitude questionnaire. In the second section, we summarise the results of applying the NPT to the inductive derived themes/subthemes (*stage 2*) and responses to the NoMAD questionnaire.

## Results

### Participants

Twenty-one participants completed an interview and 211 participants completed at least one questionnaire. Participant demographics are in Table 1. All interviewees and 71% (*n* = 149) of questionnaire participants were involved in managing and/or delivering FREED.

**Table 1:** Demographic characteristics of interview and questionnaire participants

	Interview (*N* = 21)	Questionnaire (*N* = 211)
**Age (%, n)**		
18–34 years	52% (11)	37% (77)
35–55 years	43% (9)	52% (110)
> 55 years	0% (0)	10% (21)
Missing	5% (1)	1% (3)
**Gender (%**, ***n*)**		
Female	90% (19)	85% (179)
Male	10% (2)	13% (28)
Other gender identities	0% (0)	0% (0)
Missing/Prefer not to say	0% (0)	2% (4)
**Profession (%**, ***n*)**		
Doctor	0% (0)	7% (14)
Psychologist	43% (9)	27% (57)
Nurse	29% (6)	24% (51)
Psychological therapist/Psychotherapist/Counsellor	10% (2)	12% (25)
Occupational Therapist	5% (1)	6% (13)
Dietician	0% (0)	6% (12)
Social Worker	0% (0)	3% (6)
Support Worker/Assistant Psychologist	10% (2)	10% (21)
Administrative	0% (0)	2% (4)
Team Lead/Manager	0% (0)	1% (1)
Other: Clinical	5% (1)	0% (0)
Other: Non-clinical	0% (0)	2% (4)
Missing	0% (0)	1% (3)
**Months working with FREED (%**, ***n*)**		
0–3 months	14% (3)	93% (197)
4–8 months	38% (8)	3% (6)
9–12 months	5% (1)	1% (2)
13–16 months	5% (1)	0% (0)
17–20 months	0% (0)	0% (0)
21–24 months	0% (0)	0% (0)
25 months or more	33% (7)	1% (1)
Missing	5% (1)	2% (5)

*Note* Percentages were rounded to the nearest integer

### Stage 1: inductive thematic analysis and attitude questionnaire

Six overarching themes and 15 subthemes were generated in the inductive analysis (Table 2). Means and *SD*s for attitude questionnaire subscales are in Table 3 (see Additional file 5 for item-level results) and the results of the MLMs in Table 4. First, we summarise the questionnaire results and then the qualitative subthemes. Where appropriate, responses to the specific questionnaire items have been embedded within the qualitative description.

Overall, ‘positive attitude’ and ‘the importance of early intervention for EDs’ items were rated highly before the FREED training, suggesting that clinicians tended to agree/strongly agree with these statements at the outset. In contrast, there was more indecisiveness for ‘negative attitude’ items with a trend to slightly disagree with some items obtaining an average rating of 4 (‘undecided’). These quantitative findings are largely reflected in three qualitative subthemes, namely, *“Hope and enthusiasm: Making sense of early intervention and FREED”*, *“Conflicting feelings: Eligibility and concerns about non-FREED patients”* and *“Capacity and competing demands”*. These subthemes highlight the high level of enthusiasm and positive perceptions of early intervention on the one hand and the reservations and concerns on the other. The training led to significant improvement in positive but not negative attitudes, which were maintained at the 3-month follow-up. Beliefs about knowledge and skills to deliver early intervention were moderately high before training and obtained the largest training-related improvements. This moderately high rating of perceived knowledge and skills at the outset does not fully align with the *“Self-efficacy: Experience, stress, and resilience”* subtheme, where clinicians said that it took time to understand and develop confidence with FREED. The training related improvements are reflected in the positive views of the training outlined in the “*Practical and ongoing training*” subtheme.

**Table 2:** Overarching themes and subthemes

Theme	Sub-themes
Patient	Patient engagement
	Patient complexity and comorbidities
Clinician	Hope and enthusiasm: Making sense of early intervention and FREED
	Conflicting feelings: Eligibility and concerns about non-FREED patients
	Self-efficacy: Experience, stress, and resilience
The service model	Flexibility and structure
	Champion as invaluable
	Meeting people where they are at: Care package and resources
Implementation strategy	Practical and ongoing training
	Being part of something bigger: The FREED Network
Service/team	Capacity and competing demands
	Compatibility and integration
	An open dialogue: Sharing and involvement
Wider system	Broader system of care
	Coronavirus diseases 2019 (COVID-19)

*Notes* FREED = First Episode Rapid Early Intervention for Eating Disorders

**Table 3:** Mean rating and standard deviation for each subscale on the attitude questionnaire at pre-training (Time 1), post-training (Time 2), and 3-month follow-up (Time 3)

Questionnaire subscales	Questionnaire completion time point		
	Time 1: Pre-training	Time 2: Post-training	Time 3: 3-month follow-up
Attitudes			
Positive^a^	5.96 (0.71) [*n* = 185]	6.25 (0.58) [*n* = 152]	6.16 (0.65) [*n* = 114]
Negative^a^	3.42 (0.95) [*n* = 185]	3.25 (0.99) [*n* = 152]	3.33 (1.03) [*n* = 114]
Knowledge/Skills^a^	5.42 (1.06) [*n* = 183]	6.12 (0.71) [*n* = 151]	6.07 (0.80) [*n* = 114]
Importance of Early Intervention^b^	4.66 (0.46) [*n* = 182]	4.70 (0.45) [*n* = 151]	4.72 (0.41) [*n* = 114]

^a^Rated on 7-point Likert scale ranging from ‘Strongly Disagree’ to ‘Strongly Agree’

^b^Rated on 5-point Likert scale ranging from ‘Not Important’ to ‘Absolutely Essential’

**Table 4:** Multi-level models for the change in attitude scores from pre-training (Time 1) to post-training (Time 2) and 3-month follow-up (Time 3)

Outcome	ICC	Predictors	b (SE)	β (SE)	t	df	p
Positive Attitudes	0.38	**Time: Linear**	**1.88 (0.57)**	**2.84 (0.86)**	**3.31**	**241**	**<0.005**
		**Time: Quadratic** ^**a**^	**−1.79 (0.49)**	**−2.70 (0.73)**	**−3.68**	**241**	**<0.001**
Negative Attitudes	0.64	Time: Linear	−0.49 (0.69)	−0.50 (0.70)	−0.71	241	0.48
		Time: Quadratic^a^	0.98 (0.57)	0.99 (0.57)	1.73	241	0.08
Knowledge/Skills	0.31	**Time: Linear**	**5.79 (1.08)**	**6.12 (1.43)**	**5.36**	**239**	**<0.001**
		**Time: Quadratic** ^**a**^	**−3.74 (0.56)**	**−3.95 (0.59)**	**−6.70**	**239**	**<0.001**
Importance of Early Intervention for Eating Disorders	0.55	Time: Linear	0.33 (0.32)	0.73 (0.72)	1.02	239	0.31
		Time: Quadratic^a^	0.03 (0.32)	0.06 (0.71)	0.08	239	0.94

*Note* Significant predictors are indicated in bold. ^a^Negative quadratic values indicate concave shaped relationship, whereas positive values indicate convex

### Patient

#### Patient engagement

Patient engagement was identified as a facilitator, whereas ambivalence was a barrier. Fifteen interviewees reported improved engagement for FREED patients and between 88 and 96% (varied by time point) of the questionnaire participants agreed that early intervention would improve treatment uptake. FREED was perceived as contributing to this engagement by providing a “*first positive experience*” with services (e.g., active engagement, rapid access, flexibility and providing hope for recovery). The 48-hour engagement call was perceived as a particularly valuable and easy aspect of the model to implement.

##### P010:

*“We work with a lot of young people who are ambivalent about change, so that early engagement call is most integral to what we do”*.

However, six interviewees reported a notable level of early disengagement or no improved engagement. Specifically, intervening very early, before someone was ready, was thought to result in early disengagement in some cases.

##### P016:

*“…we also had the experience of people who felt it was almost too early an intervention that everything happened too quickly […] but I think the feedback that we’ve had from them both directly and indirectly is for those people who maybe didn’t engage at the start that had a very good first positive experience of treatment […] knowing what was going to be on offer and what the options were so even if they decided that at that particular time that they weren’t quite ready, some of them have come back since”*.

#### Patient complexity and comorbidity

Clinicians spoke about patients feeling like a *“FREED patient”* (young and limited experience with services). However, in some cases, especially when patients presented with comorbidities, there were questions around appropriateness of FREED vs. other interventions, e.g., *“Do you go ahead and diagnose someone with an eating disorder and take them into treatment when it’s been there three months […] or do we understand this more in the context of stress”* (P019). A thorough evaluation of the function of the ED behaviours at the outset was seen as important to ensure patients were given the right treatment. Most questionnaire participants did not see FREED as leading to over-diagnosis: 65–73% disagreed that early intervention would result in the overtreatment of mild eating, weight or shape concerns and 78–84% disagreed that it was best to adopt a “watch and wait” approach.

### Clinician

#### Hope and enthusiasm: making sense of early intervention and FREED

##### P005:

“*The team were really, really enthusiastic about it*”.

The buy-in and enthusiasm amongst clinicians and senior staff were critical for the successful implementation of FREED. FREED was perceived as important across all EDs and services because of the expectation that it would improve outcomes and recovery, and reduce the intensity of treatment, and impact on the person’s life. These beliefs were core to how clinicians made sense of early intervention and were largely reflected in the questionnaire results. Almost 100% of questionnaire participants agreed that early intervention would improve outcomes and was important or absolutely essential. Most participants also agreed that early intervention would reduce disruptions to life and the burden on family, friends or carers. However, buy-in varied across the interviewed services with some clinicians being more cautious and sceptical of FREED.

Key enthusiastic individuals were driving FREED forward and using a range of activities to facilitate and maintain buy-in. The evidence supporting FREED, the observed impact of FREED on patients, and positive patient feedback were contributors to the narrative of hope around the model and were highly rewarding for clinicians. Clinicians valued the shift from solely focusing on physical parameters and chronicity to a pro-active, flexible, and early intervention-orientated culture.

##### P004:

*“The clinicians have really enjoyed working with it […] seeing improved outcomes for FREED patients means they’ve all got people on their caseload who are doing well”*.

#### Conflicting feelings: eligibility and concerns about non-FREED patients

Clinicians were uncomfortable knowing that some patients were not receiving early intervention, and with the message implied by FREED, i.e., that recovery will be more difficult in later stage illness. Similarly, some worried that FREED would negatively impact non-FREED waiting lists. On average, questionnaire participants were ‘undecided’ about whether early intervention would increase the wait for other patients. Interviewees also reported that FREED can be seen as *“light work”* relative to standard treatment. These concerns can create tensions within teams, especially when the waiting lists were under pressure.

##### P003:

“*Worries about impact on the rest of the waiting list and how it might negatively impact non-FREED patients can put people off*”.

Many expressed a desire to expand the age range for FREED but also recognised it as targeting resources at a peak risk period and allowing for treatment to be tailored to developmental stage.

##### P007:

“I think sometimes that it would work for a lot more people, not just under 25s, so it’s a hard one ‘cause I’m torn ‘cause I see it really working and I get why we have to do it for that, but I also wish the whole service was early intervention”

Teams who expanded or removed the upper age limit (25 years) either revised back down due to capacity issues or felt that the over 25s did not benefit from FREED in the same way. In contrast, there was no strong desire to change the duration of illness criterion because it was seen as evidence based. The main challenge with the duration of illness criterion was assessing and calculating it.

FREED having a positive impact beyond FREED patients helped counter some of the concerns around the model. Specifically, FREED principles and guides were found to be helpful for non-FREED patients, FREED enabled greater investment and expansion of services, and, in the long-term, FREED was perceived as freeing up resources for the entire service. Similarly, most questionnaire participants agreed that early intervention would reduce the long-term economic cost of EDs and disagreed that it diverts valuable resources away from longer-term EDs.

##### P009:

*“…if we can help these people get them well sooner and reduce the risk that they might relapse that eventually is gonna mean we do have more resources for people who are who tend to be more chronic or might need a higher level of care”*.

#### Self-efficacy: experience, stress, and resilience

Clinical experience with EDs and FREED, people’s confidence in their and others’ ability to implement the model, and stress and resilience were distinct but overlapping barriers and facilitators for FREED. Clinicians new to EDs found adopting FREED easier, whereas those with many years of experience and pre-existing caseloads found the change more difficult. However, more experienced clinicians reported that seeing the detrimental impact of EDs over many years increased their motivation to implement FREED. Clinician stress and anxiety on the one hand, and their resilience on the other, can impact the implementation of FREED. It took time for clinicians and the wider team to understand the model and gain confidence in implementing it.

##### P015:

“*Initially when I heard about it, I was a bit anxious about it”*.

### The service model

#### Flexibility and structure

Clinicians valued the clear structure and standardised model, they found that it kept them focused and legitimised and enabled the implementation of early intervention. Equally, if not more important to clinicians, was the adaptability and flexibility of the model. The ability to adapt parts of the model to fit the local context was a key driver in adoption and implementation. Clinicians who were flexible and focused on finding adaptive solutions also facilitated the model. The relationship between the FREED model and flexibility/creativity was reciprocal. FREED pushed and enabled teams and clinicians to be more flexible, which in turn facilitated the implementation of FREED.

##### P001:

*“FREED has allowed me the freedom”*.

#### Champion as invaluable

##### P014:

“*It’s been essential; I don’t think you could do it without the FREED Champion*”.

Having a dedicated and enthusiastic FREED Champion within the team, was identified as crucial for getting FREED set up, integrated, and sustained. The Champion was a designated person for FREED-related queries and support and provided detailed management and oversight of the pathway. However, the Champion’s role was described as demanding, and required support from senior staff and a FREED mini team.

##### P009:

*“I think FREED Champions work really hard and they do a lot of juggling actually […] I think when a lot of your responsibility is doing these engagement calls you have to hold a lot more people in mind…”*.

#### Meeting people where they are at: care package and resources

The care package and adapting treatment to emerging adults was valued by clinicians and perceived as beneficial for patients. Clinicians found the care package easy to use because the topics were relevant and/or familiar. However, family involvement was described as more challenging because it depends upon the family’s willingness and ability to engage. Other barriers for using the care package were knowing how and remembering to integrate these into treatment. Prompts, reminders, and the online FREED materials were highly valued and supported clinicians to use the care package, particularly for engaging with young people.

##### P005:

“*All of the materials that we get from that, I think that’s really crucial in driving it, so that’s absolutely, that’s a facilitator*”.

### Implementation strategy

#### Practical and ongoing training

The FREED training was described as helpful and inspiring, especially practical tasks, such as role playing and discussions within and between services, e.g., *“I really liked the fact that there was a lot of experiential exercises”* (P019). This is reflected in the questionnaire responses, whereby the training significantly improved positive attitudes, NoMAD scores, and knowledge and skills. Nevertheless, more training was desired, particularly refresher training, calculating DUED, managing early disengagement, and integrating the care package. The implementation support by SLaM/KCL/AHSNs and the Champion providing ongoing training at each site were perceived as vital components of the training.

#### Being part of something bigger: the FREED network

Being part of a wider initiative (i.e., the FREED Network) contributed towards how important the work felt and made FREED easier to *“sell”*. The FREED Network and implementation supervision provided a supportive space for sharing successes and challenges, learning, and problem-solving.

##### P014:

“*It’s nice to know that other people are experiencing the same things and it’s really easy to drop an email to people and ask for advice*”.

Sharing experiences with local services and at conferences, was important for *“taking FREED off the pedestal”* and facilitating the spread of the model. The FREED Network data collection and feedback process were valued and created a degree of peer accountability but were experienced as labour-intensive and challenging with limited resources.

##### P010:

*“…even just ‘cause like I said sending in the data every couple of months it keeps us on track […] I think it’s very easy for a service like ours to slip off and just focusing on the more chronic end […] the challenging aspect would be the initial setting up for me [and] getting everyone to fill in the ROMS [Routine Outcome Measures]”*.

### Service/team

#### Capacity and competing demands

##### P008:

“*Because obviously it comes down to the capacity*”.

Staff capacity and time were the most frequently mentioned barrier and facilitator to implementing FREED. Almost all interviewees expressed concerns about capacity regardless of whether they were currently facing capacity issues. Five teams expressed capacity-related difficulties implementing the model, especially the treatment wait time target. Questionnaire participants were also largely undecided or agreed that early intervention would increase the demand on teams. Interviewees reported that the FREED model can drift and become less of a priority over time because of competing demands. An enthusiastic Champion, a mini team, and the FREED Network were seen as working against this drift. Several strategies were used to manage capacity-related issues, including: (1) providing evidence-based individual treatments in groups; (2) flexibly and carefully balancing FREED and non-FREED caseloads; (3) low-level psychoeducational support; and (4) extending the waiting time targets.

#### Compatibility and integration

Compatibility (‘fit’) between FREED and the clinician and service as well as integrating FREED into service processes, paperwork, resources, meetings, and culture were facilitators for FREED because it made the model easier to use.

##### P015:

“*It’s part and parcel of the fabric of what we do, so we use it, and we implement it, and I don’t know how much we overly think about it*”.

Streamlining referral processes was particularly important to ensure that the referrals were received by the FREED team as quickly as possible. Protected time to deliver FREED, especially for the Champion, was also crucial for implementing the model. Conversely, differences between FREED and the standard way of working and poor integration did sometimes cause tensions and make FREED difficult to deliver.

#### An open dialogue: sharing and involvement

Sharing information, involvement in decision making, and encouraging people to reach out if they had ideas or questions created a shared and open dialogue around FREED, which facilitated its use. The FREED huddles, monthly supervision, and dedicated time in other meetings were important avenues for facilitating information sharing, and problem-solving.

##### P012:

*“…involve more people in the team, and talk about it more in our wider team, so then they feel involved and have an understanding of what FREED is”*.

Actively involving the wider service was particularly important when using a FREED ‘mini’ team because it can, at times, create a *“split”* within the ED service. A ‘whole team’ approach to FREED can also be used to guard against this *“split”* within the team (i.e., everyone in the ED service is involved with managing and/or delivering FREED). However, for a whole team approach to be successful, a considerable amount of time is needed to gain and maintain buy-in and integrate FREED into the whole service.

### Wider system

#### The broader system of care

Poor awareness of EDs and FREED amongst referrers (e.g., primary care) was a prominent barrier to receiving appropriate and early referrals for FREED, especially for newer sites and services who historically had not accepted milder early intervention cases. Wider awareness of EDs and FREED at educational institutions, amongst healthcare professionals and the public was considered as essential for enabling the earliest identification of EDs. FREED associated awareness raising activities were highly valued by clinicians and perceived as a core part of the early intervention work.

##### P017:

“*… the biggest barriers so far is getting the referrals through*”.

#### COVID-19

COVID-19 was primarily a barrier to implementing FREED but did bring about some positive changes (e.g., virtual appointments providing greater flexibility and reduced travel time). COVID-19 disrupted and restricted services (and therefore FREED), which reduced capacity and dramatically limited services. This period was difficult for clinicians because of the elevated risk, changes in working, reduced team communication, and therapy itself was seen as more challenging online. It was difficult to keep early intervention going, and FREED became less of a priority as other COVID-19 related issues took precedence.

##### P013:

“*Early intervention has had to take a little bit of a backseat in that sense just because of how sparse we are with resources*”.

### Stage 2: Normalisation Process Theory

The qualitative summary of the NPT mechanisms underlying each subtheme is outlined in Table 5. Means and *SDs* for each NoMAD questionnaire subscale at each time point are in Table 6 (see Additional file 5 for item-level results) and the results of the MLMs in Table 7.

The qualitative and quantitative data suggest that FREED was largely normalised or normalising in many services. In other words, there was a high level of NPT ‘work’ taking place across the subthemes and most NoMAD scores were high. Coherence in terms of understanding the model and its value was high amongst interviewees and questionnaire participants. The FREED training, a key coherence building activity, led to significant improvements in the coherence subscale. However, the qualitative data suggest that coherence was less well-developed for newer sites, especially for the care package and wider team. Cognitive participation (i.e., creating and sustaining engagement) was the highest rated NoMAD subscale, which was reflected in the qualitative data. Specifically, interviewees reported that there were key individuals enrolling and engaging others in FREED work (typically, but not always, the FREED Champion). The FREED Champion, training, network, and mini team were also seen as legitimising FREED and maintaining engagement over time. In keeping with this, the training was found to significantly improve cognitive participation. The ‘work’ associated with collective action was evident across all subthemes as clinicians enacted and integrated FREED into relations, interactions, and contexts. While applying the collective action construct, it became apparent that it was the main NPT mechanism by which established sites differed from newer sites. This aligns with the finding that collective action was the lowest rated NoMAD subscale as many of the questionnaire participants were relatively new to FREED. While the training led to improvements in some features of collective action (e.g., confidence and skills), others, such as the perception of sufficient capacity, were less impacted. Insufficient capacity was the main factor inhibiting normalisation, even when FREED was well-integrated into other aspects of the team. Changes in capacity and fluctuating demand required teams to continually appraise and re-configure the structure and functioning of FREED. All interviewees engaged in formal (data) and informal (practice experience) reflexive monitoring of what was and was not working and whether FREED was worthwhile. It was most evident in the ‘*Being part of something bigger: The FREED Networ*k’ and ‘*An open dialogue: Sharing and involvement*’ subthemes. Responses to the NoMAD questionnaire suggest that the training increased clinician capacity to engage in reflexive monitoring.

**Table 5:** Normalisation Process Theory mechanisms underlying each theme and subthemes

Themes/Subthemes	Normalisation Process Theory Mechanisms			
	Coherence	Cognitive Participation	Collective Action	Reflexive Monitoring
Patient				
Patient engagement	- The patients’ understanding of the benefits of early intervention was an important for individuals to engage with the model. - The active outreach and engagement work were valued by clinicians and were seen as important for patient coherence.	- Outreach, the engagement call, and emphasising the importance of early intervention enrols patients in FREED work.	- Engagement calls were easy to integrate but depends on the relation/interaction with patient and/or referrer. - Rota system used in some teams to distribute engagement calls.	- Individual clinicians were engaged in appraisal work regarding the impact of FREED on motivation and engagement.
Patient complexity and comorbidities			- Difficulties determining suitability for FREED. - Individual and collective work (i.e., thorough evaluation and team discussions) to determine and develop confidence in suitability.	
**Clinician**				
Hope and enthusiasm: Making sense of early intervention and FREED	- There was a high degree of individual and collective understanding of FREED and its value in FREED teams. - The potential benefits of FREED to patients were core to how clinicians made sense of FREED. - There was a high degree of personal alignment and internalisation of the objectives of FREED amongst clinicians. - Assessing the evidence-base was a key mechanism in how clinicians attribute value to FREED. - Comparison of FREED against standard illness prioritisation procedures built coherence towards the model.	- Key enthusiastic individuals drive FREED forward using a range of activities to create and maintain ‘buy-in’.	- Clinician and senior staff supporting the adoption of FREED was central to implementation and the distribution of resources.	- Appraisal of the evidence-base and the observed impact on patients and the team was used to evaluate the worth of FREED.
Conflicting feelings: Eligibility and concerns about non-FREED patients	- Individual and collective concerns regarding the impact on waiting lists and non-FREED patients were key barriers. - Wider team did not always value all aspects of FREED (i.e., perceived as ‘privileged’ and ‘light’ work). - Most clinicians perceived FREED as beneficial for all ages. Equally, the age eligibility criterion was understood as pragmatic and enabled tailoring to developmental stage. - The value of FREED was perceived to extend beyond FREED patients.		- Some services adapted the eligibility criteria to align with their service and beliefs. - Difficulties determining duration of an untreated eating disorder due to confidence/skills, and clarity of information from patient.	- Ongoing clinician appraisal of the broader impact of the model (i.e., impact on non-FREEDs, wider service). - Clinicians re-configured the eligibility criteria and formally (data) and informally (personal experience) appraised the change.
Self-efficacy: Experience, stress, and resilience	- Greater experience in EDs increases the internalisation of FREED as important and needed.		- Individual skills and belief about skills and capacity to implement FREED impacted the implementation. - Continued investment and engagement with FREED builds skills and confidence around the model over time. - Individuals with pre-existing caseloads and many years in EDs are required to do more work to integrate FREED into their existing practice. - Active support to manage stress/anxiety provides individuals with the resources to engage in FREED work.	- Ongoing appraisal regarding oneself and other’s ability to understand and use the model.
**The Service Model**				
Flexibility and structure	- Structure enables clear understanding of the specific tasks and steps needed to implement FREED. - An understanding of how FREED compares to standard practice was needed to adapt it to the local context. - The flexibility around the model was valued.		- There was individual and collective work taking place to adapt FREED to ‘fit’ the local context (e.g., sharing the Champion responsibilities, ‘whole team’ approach to implementing FREED) – largely undertaken by senior staff and FREED Champion.	
Champion as invaluable		- Champion as designated individual that drives FREED forward, creates, and maintains engagement, and enrols others in FREED work.	- Champion distributes and manages the work and resources needed to implement FREED. - Champion supports ongoing training and skill development to enable clinicians to implement FREED (also relevant to the Practice and ongoing training subtheme). - Insufficient capacity for Champion to complete all tasks. Sharing and delegating Champion tasks and responsibilities is often needed.	
Meeting people where they are at: Care package and resources	- Tailoring treatment perceived as beneficial and valued. - Some difficulties understanding how and when to integrate care package adaptations into standard treatment. - Some unawareness of care package components (typically at outset and in wider team).	- Tailoring treatment and having resources available online engages clinicians and patients into FREED work.	- Work was required to adapt standard treatment to accommodate FREED adaptations. - FREED-related materials (e.g., tracker template), prompts, reminders, and using different communication methods made FREED easier to integrate into work. - The interaction between the patient’s life stage and adaptations can make the adaptations easy (e.g., relevance) and difficult (e.g., family involvement for students) to use.	
**Implementation Strategy**				
Practical and ongoing training	- Training and its continuation as key to developing individual and collective understanding of FREED and its benefits.	- Training supports initiation and legitimisation of FREED.	- Sufficient training was undertaken to develop the skills to implement FREED, but more and ongoing training was desired. FREED Champion was key for ongoing training and skill development in teams.	
Being part of something bigger: The FREED Network	- Network enabled teams to work together to make sense of FREED and its implementation. - Wider investment and interest lead to greater internalisation of the importance of FREED. - Conferences as key medium to share information and “take FREED off the pedestal”.	- Network and data feedback create a broad community of practice that legitimises and maintains engagement.	- Implementation supervision and ongoing evaluation contribute towards accountability and confidence in using the model. - Data collection work shared with/delegated to assistant psychologists, support workers, and administrators.	- Formal and informal appraisal during implementation supervision and data feedback to evaluate whether FREED and its components are working and worthwhile.
**Service**				
Capacity and competing demands	- Concerns regarding capacity at the outset and over time can impact value attributed to FREED.	- Champion, mini team, and Network identified as important for maintaining momentum and engagement amongst competing demands.	- Insufficient resources allocated to implement FREED in some but not all teams. - Individually and collectively adapting mental and material resources to address capacity issues.	- Ongoing individual and communal appraisal around capacity and the re-configuration of FREED and treatment as usual as capacity fluctuates.
Compatibility and integration	- Developing an understanding of how FREED differs from standard practice was done to allow for integration work. - At the outset, FREED was sometimes perceived as “special” and very different from standard practice, which was a barrier, but this changed over time as it became integrated.	- Integration and protected time supported the enrolment, legitimisation, and sustainability of FREED.	- Compatibility with the existing service and clinician values and practice was a facilitator. - Relational and contextual integration through integrating into service processes and procedures, culture, and resources (e.g., protected Champion time and meetings). - Limited integration with wider team can disrupt working relations and FREED. - Carefully balancing and integrating FREED and non-FREED work was important.	- Dedicated FREED huddles and integrating FREED into discussion in general meetings was used to appraise FREED work. - Clinicians appraised and re-configured to overcome integrational barriers.
An open dialogue: Sharing and involvement	- Involvement and an open dialogue allowed teams to work together to develop a shared understanding of the model, its benefits, and to address concerns.	- Active involvement and creating an open dialogue initiate and enrol clinicians in FREED work. - Mini team enables ongoing engagement and maintenance of the model.	- Subtheme included the interactional work people do around FREED to develop accountability and confidence in the model. - Allocated time in meetings to enable interactional work to take place. - FREED work distributed amongst the entire team or mini team. - FREED can disrupt working relations/create a divide in the service.	- Communal appraisal of the functioning, and problems around FREED was an important facilitator. - Re-configuring the structure of FREED, i.e., mini vs. whole team approach, following appraisal and then appraising the value of this re-configuration.
**Wider System**				
Broader system care	- A wider shared understanding (e.g., public, healthcare services) of EDs and FREED is needed for early identification but was not always present. - Understanding of outreach work as a core responsibility in early intervention and a valued part of FREED.	- Identification and enrolment of referrers at the outset is needed to ensure successful implementation.	- Funding/resources needs to be obtained quickly from the broader system (e.g., commissioners) to enable implementation. - Relational work with educational institutions and referrers was taking place to ensure early identification and appropriate referrals.	- Clinicians engaged in appraisal work regarding the referral pathways and processes into the service to ensure the earliest identification.
Coronavirus diseases 2019 (COVID-19)	- FREED still perceived as important; however, less important relative to pressing COVID-19 demands.		- COVID-19 disrupted interactional and relational work. Clinicians and patients required to re-establish relations and implement FREED in the context of COVID-19. - Clinicians worked to re-operationalise and maintain FREED in altered circumstances (e.g., virtual appointments).	- Clinicians were routinely engaged in informal appraisal of the positive and negative impacts of virtual working.

**Table 6:** Mean rating and standard deviation for each subscale on the NoMAD questionnaires at pre-training (Time 1), post-training (Time 2), and 3-month follow-up (Time 3)

Questionnaire subscales	Questionnaire completion time point		
	Time 1: Pre-training	Time 2: Post-training	Time 3: 3-month follow-up
NoMAD			
Coherence	4.00 (0.50) [*n* = 179]	4.22 (0.49) [*n* = 154]	4.23 (0.48) [*n* = 115]
Cognitive Participation	4.43 (0.47) [*n* = 181]	4.53 (0.46) [*n* = 153]	4.56 (0.42) [*n* = 115]
Collective Action	3.95 (0.54) [*n* = 179]	4.12 (0.49) [*n* = 152]	4.12 (0.63) [*n* = 115]
Reflexive monitoring	4.11 (0.51) [*n* = 180]	4.29 (0.49) [*n* = 152]	4.32 (0.47) [*n* = 115]

*Note* All items rated on 5-point Likert scale range from ‘Strongly Disagree’ to ‘Strongly Agree’. NoMAD = Normalization MeAsure Development

**Table 7:** Multi-level models for the change in NoMAD scores from pre-training (Time 1) to post-training (Time 2) and 3-month follow-up (Time 3)

Outcome	ICC	Predictors	b (SE)	β (SE)	t	df	p
Coherence	0.36	**Time: Linear**	**1.98 (0.40)**	**3.95 (0.80)**	**4.96**	**240**	**<0.001**
		**Time: Quadratic** ^**a**^	**−0.99 (0.34)**	**−1.98 (0.68)**	**−2.92**	**240**	**<0.005**
Cognitive Participation	0.51	**Time: Linear**	**0.92 (0.35)**	**2.01 (0.76)**	**2.63**	**239**	**<0.01**
		Time: Quadratic^a^	−0.33 (0.31)	−0.70 (0.68)	−1.06	239	0.30
Collective Action	0.50	**Time: Linear**	**1.46 (0.45)**	**2.64 (0.81)**	**3.26**	**237**	**<0.005**
		**Time: Quadratic** ^**a**^	**−1.15 (0.34)**	**−2.08 (0.61)**	**−3.40**	**237**	**<0.001**
Reflexive Monitoring	0.42	**Time: Linear**	**1.79 (0.39)**	**3.58 (0.79)**	**4.50**	**239**	**<0.001**
		**Time: Quadratic** ^**a**^	**−0.71 (0.35)**	**−1.42 (0.71)**	**−2.00**	**239**	**<0.05**

*Note* Significant predictors are indicated in bold. ^a^Negative quadratic values indicate concave shaped relationship, whereas positive values indicate convex

## Discussion

The aim of this study was to understand clinician attitudes towards early intervention for EDs and FREED, implementation processes and barriers and facilitators to implementation at the start of a national scaling process. The NPT was used as a conceptual lens to provide further insights into the implementation and embedding of FREED. The questionnaire results suggest that even before attending the training clinicians were highly positive towards early intervention and could see the potential value of FREED (coherence). Participants also agreed that there were key people driving FREED forward, that FREED was a legitimate part of their role and were open to working with colleagues in new ways (cognitive participation). All of this provides a highly receptive context for the implementation of FREED. This high ‘buy-in’ and engagement were also evident in the qualitative data and identified as a key facilitator for implementing FREED, especially when faced with obstacles and challenges. However, questionnaire and interview participants were concerned about the impact on capacity and felt uncomfortable knowing that other patients were not receiving treatment as quickly. These concerns, at times, led to tensions within teams and disengagement with the model, particularly early in the implementation. Previous studies on clinician attitudes towards early intervention in mental health have reported similar findings. Specifically, that early intervention was seen as useful and important, but clinicians were concerned about resources and the implications for patients not eligible for early intervention [20, 42, 43]. However, the presence and impact of these views on the implementation of early intervention for EDs has not been widely evaluated. The findings of this study resonate strongly with the recent qualitative study by Hyam et al. [18] of the views and experiences of AHSN implementation leads during the national scaling of FREED. The replication of results with differing samples and methodological approaches strengthens the conclusions of both studies.

Several features of the service model and its implementation were valued by clinicians, impacted NPT mechanisms, and facilitated implementation. These included: the evidence-base, data collection and feedback; FREED Network; Champion; training; and observing the impact of FREED on patients. While the training was effective at improving positive attitudes and NPT mechanisms, it did not impact concerns about capacity and waiting times for other patients; also, more training was desired. The FREED Champion played a vital role in cognitive participation and integrating and embedding the model. The Champion was supported in this work by the ‘mini’ team, FREED Network, and central implementation support. Creating an open dialogue around FREED, which was largely accomplished by the Champion, was important for all the NPT mechanisms as it allowed people to make sense of, engage with, embed, and reflect on FREED. However, the Champion could not do this on their own and required the support of senior staff and the wider team to enable them to fully embed FREED. This aligns with research demonstrating that change efforts are more likely to be successful if there are multiple layers of champions across different levels within an organisation [44]. The compatibility, flexibility, and the integration of FREED into the services facilitated its uptake and implementation. This is in accordance with evidence on the importance of innovation-system fit and the co-evolution of the intervention and context over time [45, 46]. Other valued features of the model were the active outreach and engagement (e.g., engagement call), the care package and materials, and the model’s clear structure.

There were also some areas of concern that warrant attention in the future implementation of FREED. Capacity, which is generally an issue in publicly funded health services, was one of the most prominent concerns for participants and prevented the normalisation of FREED. Capacity issues were made worse by the COVID-19 pandemic. The FREED data collection and treatment wait time target were perceived as challenging to implement because of capacity. This finding is reflected in the high level of missing data in the national FREED dataset and the low level of adherence to the treatment wait time target [47]. While the wait time targets are not implemented punitively/strictly, the long-term impact of consistently not meeting a target on clinician morale needs to be monitored in the future. There were also concerns that FREED could impact the standard waiting list and patients not eligible for the service. It is important that any decision to continue to scale the model evaluates potential unintended consequences on the rest of the service. Potential solutions for capacity issues include additional investment, brief or groups treatments (e.g., CBT-T;^48^), and task-sharing interventions (e.g., peer support workers;^49^). Peer support workers are a promising avenue for FREED as peers can instil hope and draw on the power of relatable and reciprocal relationships [50].

Another prominent barrier to early intervention was quickly receiving appropriate and early referrals from the team and wider healthcare system. In the team, it was important to develop processes for quickly allocating FREED patients. Outside the team, clinicians reported using awareness raising activities with health and educational professionals to increase early referrals. Monitoring the success of these activities would be of interest, especially as evidence for interventions to increase the early detection of EDs is mixed [51, 52]. Relatedly, clinicians disagreed that early intervention would result in over-treatment and a “wait and watch” approach should be used. However, a comprehensive evaluation of the function of the ED behaviours was seen as important to ensure suitability for FREED.

### Limitations

Participants in this study were clinicians attending FREED training or already delivering FREED. Most were also psychologists or nurses. Therefore, they are unlikely to be representative of all clinicians working in ED services in England and other countries. All interviewed clinicians were also early adopters of FREED. Early and late adopters can differ significantly in attitudes, resources and context [53]. The attitude questionnaire was not well-established or validated, and some subscales of both questionnaires had less than desirable internal consistency (α ≥ 0.7), which may have impacted the validity of those subscales.

## Conclusion

This study provides valuable insights into the clinicians’ perspective of early intervention for EDs and FREED and the complex interaction between attitudes, NPT mechanisms, implementation and context. Clinicians were largely positive towards early intervention for ED and FREED but also concerned about capacity and the impact on patients not eligible for the service. Insufficient capacity was a major barrier that needs addressing during the next phase of implementation. It is essential that the acceptability, feasibility, and experiences of FREED clinicians continue to be evaluated as it is further scaled. It takes time for new ways of working to become fully embedded and part of routine practice. This process is messy and often punctuated by seemingly impassable obstacles and major setbacks [21]. Drawing on the experiences of clinicians trying to implement the model is crucial if we are to shape the evolution and implementation of FREED in a sustainable and grounded way.

### Electronic supplementary material

Below is the link to the electronic supplementary material.

Supplementary Material 1

Supplementary Material 2

Supplementary Material 3

Supplementary Material 4

Supplementary Material 5

## Data Availability

The anonymized questionnaire dataset supporting the conclusions of this article is available on the King’s Open Research Data System (KORDS) [dataset DOI: 10.18742/25498438]. The qualitative data set will not be made publicly available for privacy reasons (potential for identification).

## Abbreviations

AHSNs: Academic Health Science Networks
CBT-T: Cognitive Behavioural Therapy - Ten
DUED: Duration of an untreated eating disorder
ED: Eating disorders
FREED: First Episode Rapid Early Intervention for Eating Disorders
KCL: King’s College London
MLM: Multi-level model
NHS: National Health Service
NoMAD: Normalization MeAsure Development
NPT: Normalisation Process Theory
RE-AIM: Reach, Effectiveness, Adoption, Implementation, and Maintenance
SLaM: South London and Maudsley
