# Supplementary material for: Clinician perspectives of the implementation of an early intervention service for eating disorders in England: a mixed method study
Source: J Eat Disord. 2024 Apr 5;12:45. doi: 10.1186/s40337-024-01000-4 (PMC10996085; doi:10.1186/s40337-024-01000-4)
Supplement: Supplementary file 1 — Supplementary Material 1 [file 40337_2024_1000_MOESM1_ESM.docx]

**Clinician perspectives of the implementation of an early intervention service for eating disorders in England: A mixed method study**

**Interview Topic Guide**

Participant background and general views and experience of early intervention for eating disorders (coherence)

1. Can you tell me a little bit about your role within the team?
   1. how long have you been in this position?
2. Could you give me a brief description of your understanding of early intervention?
3. Have you had any experience working in early intervention before FREED?
4. What are your views on early intervention for eating disorders?
   1. does the importance of early intervention differ for different eating disorders?
   2. are there any benefits or downsides of early intervention for eating disorders?

FREED initiation: coherence and cognitive participation

1. How and when were you first introduced to FREED?
   1. Was there anyone driving it forward in your team?
2. Could you give me a brief description of your understanding of the FREED model?
3. What are your views on the model? (e.g., age range, duration criterion, intervention tweaks)
4. In the NHS patients are usually prioritised based upon clinical need. In eating disorders teams this typically means that patients are prioritised according to medical urgency (e.g., low weight). In contrast, FREED patients are also prioritised due to early stage of illness. What is your opinion on this difference?
   1. Does this difference cause any tension in the team?
   2. If so, how is this tension managed?
5. Have you received any FREED training?
   1. What was your experience of the training?
      1. Did you find it helpful?
      2. Was there anything missing?
      3. Could anything have been differently?
6. Did you have any hopes and/or concerns about FREED before using it?

Implementing FREED: collective action and reflexive monitoring

1. How have you found the FREED model so far?
2. How, if at all, has working with FREED or having FREED in your team changed the way you work?
   1. How easy or difficult was it to integrate FREED into your existing work?
   2. Were any changes made to the FREED model to make it fit within your team?
   3. Has it influenced your approach and/or relationship to FREED and non-FREED patients?
   4. Has it influenced how you work with other people in your team?
3. Do you feel that your experience of FREED has been similar or different to other members of your team?
   1. What do other people in your team think about FREED?
   2. Is there a shared understanding of FREED?
   3. Are you confident in other people’s ability to use FREED?
4. Do you think that FREED has affected treatment uptake, engagement, or satisfaction?
5. Thinking about FREED as whole, what do you think were the most significant barriers and facilitators to using it?
6. How easy or difficult has it been to provide the 48-hour engagement call?
7. How easy or difficult has it been to meet the wait time targets?
8. How easy or difficult has it been to use the FREED treatment adaptations, such as increasing attention on social media and emerging adulthood?
9. How important is it or has it been to have a FREED Champion in the team?
   1. What is the most important aspect of their role?
   2. Is there anything that could or should be done differently?
10. How important is it or has it been to have and be part of the FREED Network?
11. What are the 3 best and 3 most challenging aspects of FREED?

Looking ahead

1. What factors do you think might influence the team’s ability to continue to support FREED?
2. Do you have any thoughts about how early intervention for eating disorder or FREED could be improved in the future?
3. Is there anything you wish you had known before FREED was introduced in your team?
4. I think that’s basically everything I had to ask you, is there anything else you’d like to say, or any further thoughts?

Optional COVID-19 questions

1. How has COVID-19 impacted your work and FREED?
2. Has COVID-19 impacted how important you think early intervention is?
