# Supplementary material for: Clinician perspectives of the implementation of an early intervention service for eating disorders in England: a mixed method study
Source: J Eat Disord. 2024 Apr 5;12:45. doi: 10.1186/s40337-024-01000-4 (PMC10996085; doi:10.1186/s40337-024-01000-4)
Supplement: Supplementary file 2 — Supplementary Material 2 [file 40337_2024_1000_MOESM2_ESM.docx]

**Clinician perspectives of the implementation of an early intervention service for eating disorders in England: A mixed method study**

**RE-AIM dimensions and FREED implementation strategies**

Table 1. RE-AIM dimensions and the corresponding FREED implementation strategies (adapted from Allen et al., 2020).

| RE-AIM dimension | FREED implementation strategy |
| --- | --- |
| *Reach*: The absolute number, proportion, and representativeness of the individuals willing to engage with and targeted by the service, i.e., who is the target audience and how are we reaching them? | - Key target audiences were identified (e.g., patients, families, GPs, commissioners) and materials, such as summaries of evidence and impact, were developed and tailored to each target audience, including both quantitative data and real-life stories. - Information on the FREED website (www.freedfromed.co.uk), including freely available online training modules and psychoeducation materials. - Traditional publications (e.g., blogs, news articles) and social media (e.g., Facebook, Twitter, and Instagram) used to disseminate key messages and engage stakeholders. - Establishing a “FREED Network” to facilitate communication and collaboration between all services using the FREED model, and scaling across regions. - Lived experience involvement via co-creation, and input on FREED initiatives, implementation, materials and resources, social media, and real-life stories (“FREEDom Finders”). - FREED-specific events and presentations at professional conferences, events, and seminars. - Publication of research articles and inclusion in position statements and clinical guidelines. |
| Effectiveness: The impact of the service on important outcomes, i.e., how do we know that FREED is effective? | - Formal effectiveness studies incorporating quantitative and qualitative data have been conducted from the outset. - Ongoing evaluation was built into the operational processes of the model. FREED sites share a core set of de-identified data every quarter that contributes to a national FREED data set. |
| Adoption: The absolute number, proportion, and representativeness of the settings and intervention agents (clinicians delivering the service) willing to adopt the service, i.e., how do we develop intervention agent and organisational support to deliver FREED? | - FREED readiness and equivalence assessments: an evaluation of pre-existing service characteristics and compatibility with FREED service model (e.g., evidence-based, existing service processes, enthusiasm for change), additional preparation may be needed for some sites. - Extensive stakeholder engagement and relationship building activities to develop buy-in at all levels, including service users, providers, commissioners, community stakeholders, and senior executives. Including specific efforts to develop a sense of ownership, service user involvement at all stages, and involving all stakeholders in strategic decisions. - FREED-specific implementation materials, such as a FREED business case, data collection templates and agreements, and a Champion Pack. - One-to-one implementation support from SLaM and AHSNs. - Shared regional learning and local peer leadership, e.g., FREED Champion regional leads supporting neighbouring ED services. |
| Implementation: The implementation fidelity, adaptations, and time and cost of delivery, i.e., how do we ensure that FREED is delivered properly? | - A clear training package: (1) Online training platform: initial orientation to the FREED service model, evidence, and principles. (2) Single-day in-person/virtual training delivered by the FREED team at SLaM. The training consists of presentations, concrete examples of how to apply FREED principles and processes, interactive polls/activities, role-playing, and group discussions. (3) Train-the-trainer approach, whereby trained FREED Champions and leads continue ongoing training and support locally. - Ongoing implementation support and monthly peer implementation supervision for FREED Champions. - Enthusiastic FREED Champion responsible for managing and championing the pathway. - Internal FREED-specific supervision. - FREED-specific guides, materials, resources, and videos to support implementation. - The FREED Network and regional peer support networks and collaboratives to facilitate communication, collaboration, and shared learning. - A ‘hard core, soft periphery’ approach: adherence to the core components of the model while also enabling a degree of adaptability to local pressures and needs to maximise the acceptability and fit of the model locally. - Quarterly data summary reports regarding individual site and entire Network performance. |
| Maintenance: The extent to which an intervention is sustained and/or becomes institutionalised and part of routine practice, i.e., how can FREED become standard practice and for it to be delivered in the long-term? | - Buy-in at all levels and the whole team supporting FREED. - Enthusiastic FREED Champion with “can do” attitude. - Continued engagement, cross-site learning, and collaboration through the FREED Network, FREED events and media, supervision, and data sharing and feedback. - Developing a shared sense of ownership through collaboration, shared learning, involvement in decisions, and capacity to adapt FREED. Train-the-trainer model and FREED Champion role also contribute towards model ownership. |

*Note.* AHSN = Academic Health Science Network; ED = eating disorder; FREED = First Episode Rapid Early Intervention for Eating Disorders; GP = General Practitioner; RE-AIM = reach, effectiveness, adoption, implementation and maintenance; SLaM = South London and Maudsley.
