# Supplementary material for: Clinician perspectives of the implementation of an early intervention service for eating disorders in England: a mixed method study
Source: J Eat Disord. 2024 Apr 5;12:45. doi: 10.1186/s40337-024-01000-4 (PMC10996085; doi:10.1186/s40337-024-01000-4)
Supplement: Supplementary file 3 — Supplementary Material 3 [file 40337_2024_1000_MOESM3_ESM.docx]

**Clinician perspectives of the implementation of an early intervention service for eating disorders in England: A mixed method study**

**Researcher description**

The lead researcher (KR) was a doctoral student and part of the First Episode Rapid Early Intervention for Eating Disorders (FREED) implementation and evaluation team while conducting the research study. The lead researcher’s professional background is in psychology, and she had postgraduate level training in qualitative and quantitative research methods prior to conducting the study. Before the study, the lead researcher had no experience with early intervention for eating disorders or clinician attitudes towards early intervention for eating disorders. Within the FREED implementation team, the lead researcher was largely responsible for leading the national data collection and feedback process (i.e., every 3 months they would collect data from sites, analyse the data and then provide summary reports to each site). The lead researcher also supported the FREED training and implementation supervision sessions. The lead researcher’s position carries advantages and disadvantages in relation to the research study. An advantage is that they had in-depth knowledge of the implementation of FREED and the context surrounding the implementation, which enabled a high degree of engagement with the context surrounding the participants. The lead researcher also had established relationships with some (but not all) of the participants, which allowed for a more conversational and less synthetic interaction. The drawback to having established relationships and the lead researcher’s role was that participants would sometimes specifically ask questions or talk about the data and data collection process. Another potential disadvantage of the lead researcher’s role was that participants may have been less forthcoming with negative attitudes and experiences. However, this did not appear to impact the interviews as study participants appeared to talk openly with the lead researcher about their negative views of and experiences with FREED. As outlined in the Methods section of the manuscript, specific efforts were made to highlight the importance of understanding both positive and negative attitudes and experiences to study participants as well as assuring confidentiality. Other mitigation strategies involved reflective note taking, member checking and independent coding by a second coder to ensure trustworthiness and credibility of the analysis. The second coder was an experienced qualitative researcher working in eating disorders who was independent of the FREED evaluation and implementation team at the time of the study.
