# Supplementary material for: Clinician perspectives of the implementation of an early intervention service for eating disorders in England: a mixed method study
Source: J Eat Disord. 2024 Apr 5;12:45. doi: 10.1186/s40337-024-01000-4 (PMC10996085; doi:10.1186/s40337-024-01000-4)
Supplement: Supplementary file 4 — Supplementary Material 4 [file 40337_2024_1000_MOESM4_ESM.docx]

**Clinician perspectives of the implementation of an early intervention service for eating disorders in England: A mixed method study**

**The development of the** **attitudes towards early intervention for eating disorders questionnaire**

A preliminary set of 26 potential items were developed based on unpublished qualitative data from the FREED-Up study and questionnaires from other studies evaluating clinician attitudes towards early intervention in psychosis (Rosen et al., 2012) and mental health (Ghio et al., 2015). For questionnaire development, attitudes were defined as positive or negative beliefs about the consequences of early intervention for eating disorders (EDs). Items measuring beliefs about knowledge and skills to implement early intervention approaches were also incorporated into the questionnaire because they can mediate relationship between attitudes and behaviour (Ajzen, Fishbein, Lohmann, & Albarracín, 2018). The preliminary items were rated for relevance, terminology, and phrasing by a group of ED clinicians and researchers at the South London and Maudsley and King’s College London. Items were then grouped, re-phrased and/or removed based upon this feedback. A set of 15 questions were then re-administered for further feedback before the final set of 15 questions were agreed upon in the study team. The final questionnaire consists of 6-items for positive attitudes, 5-items for negative attitudes, 2-items for beliefs about knowledge and skills, and 2-items to evaluate the importance of early intervention for EDs. Most items were rated on a 7-point Likert scale ranging from “strongly disagree” (1) to “strongly agree” (7). The items measuring the importance of early intervention for EDs were rated on a 5-point Likert scale ranging from “not important” (1) to “absolutely essential” (5).

References

Ajzen, I., Fishbein, M., Lohmann, S., & Albarracín, D. (2018). The influence of attitudes on behavior. In D. Albarracín & B. T. Johnson (Eds.), *The Handbook of Attitudes* (pp. 197-255). New York, USA: Routledge.

Ghio, L., Respino, M., Murri, M. B., Cocchi, A., Meneghelli, A., Amore, M., & Natta, W. (2015b). Attitudes of mental health workers towards early interventions in psychiatry: A national survey. *Journal of Nervous and Mental Disease, 203*(10), 756-761. doi:10.1097/NMD.0000000000000363

Rosen, A., Gorrell, J., Cornish, A., Miller, V., Tennant, C., Nash, L., & McKay, D. (2012). Promoting constructive change in the service system: A qualitative study of change in staff attitudes with the implementation of early intervention in psychosis. In K. M. Boydell (Ed.), *Hearing Voices: Qualitative Inquiry in Early Psychosis* (pp. 95-118). Toronto, Canada: Wilfrid Laurier University Press.

**Attitudes towards early intervention for eating disorders questionnaire**

In this section we want to get a better understanding of what people think about early intervention services for eating disorders (ED). Here, early intervention refers to services designed for the needs of young people with early-stage eating disorders. This does not include prevention or at-risk programmes or interventions.

Read each item carefully and indicate how strongly you agree or disagree with each statement by circling the appropriate number.

|  | Early intervention for eating disorders… | Strongly Disagree | Disagree | | Slightly Disagree | | Undecided | Slightly Agree | | Agree | | Strongly Agree |
| --- | --- | --- | --- | --- | --- | --- | --- | --- | --- | --- | --- | --- |
| 1. | will improve patient outcomes in early-stage EDs | 1 | 2 | | 3 | | 4 | 5 | | 6 | | 7 |
| 2. | will increase waiting times for other patients | 1 | 2 | | 3 | | 4 | 5 | | 6 | | 7 |
| 3. | will reduce disruption to work, education, and social relationships of early-stage ED patients | 1 | 2 | | 3 | | 4 | 5 | | 6 | | 7 |
| 4. | will result in the overtreatment of short-lived or mild eating, weight, and shape concerns | 1 | 2 | | 3 | | 4 | 5 | | 6 | | 7 |
| 5. | will reduce the long-term economic costs of EDs | 1 | 2 | | 3 | | 4 | 5 | | 6 | | 7 |
| 6. | will increase the demand on teams | 1 | 2 | | 3 | | 4 | 5 | | 6 | | 7 |
| 7. | will improve the treatment uptake of early-stage ED patients | 1 | 2 | | 3 | | 4 | 5 | | 6 | | 7 |
| 8. | will divert valuable resources away from those with more severe/enduring forms of the illness | 1 | 2 | | 3 | | 4 | 5 | | 6 | | 7 |
|  | | Strongly Disagree | Disagree | | Slightly Disagree | | Undecided | Slightly Agree | | Agree | | Strongly Agree |
| 9. | will reduce waiting times for early stage ED patients | 1 | 2 | | 3 | | 4 | 5 | | 6 | | 7 |
| 10. | will reduce the burden on family, friends, or carers | 1 | 2 | | 3 | | 4 | 5 | | 6 | | 7 |
|  |  | Strongly Disagree | Disagree | | Slightly Disagree | | Undecided | Slightly Agree | | Agree | | Strongly Agree |
| 11. | I am aware of the rationale, principles, and ways of delivering early intervention for EDs | 1 | 2 | | 3 | | 4 | 5 | | 6 | | 7 |
| 12. | It is best to adopt a ‘watch and wait’ approach for people with milder ED symptoms | 1 | 2 | | 3 | | 4 | 5 | | 6 | | 7 |
| 13. | I have the clinical skills to deliver interventions that are tailored to the illness and developmental stage of early ED patients | 1 | 2 | | 3 | | 4 | 5 | | 6 | | 7 |
|  |  | Not Important | | Slightly Important | | Moderately Important | | | Important | | Absolutely Essential | |
| 14. | How important do you consider early intervention for the following disorders? |  | |  | |  | | |  | |  | |
|  | Anorexia Nervosa | 1 | | 2 | | 3 | | | 4 | | 5 | |
|  | Other Eating Disorders | 1 | | 2 | | 3 | | | 4 | | 5 | |
