# Supplementary material for: Clinician perspectives of the implementation of an early intervention service for eating disorders in England: a mixed method study
Source: J Eat Disord. 2024 Apr 5;12:45. doi: 10.1186/s40337-024-01000-4 (PMC10996085; doi:10.1186/s40337-024-01000-4)
Supplement: Supplementary file 5 — Supplementary Material 5 [file 40337_2024_1000_MOESM5_ESM.docx]

**Clinician perspectives of the implementation of an early intervention service for eating disorders in England: A mixed method study**

**Item-level responses for attitudes towards early intervention for eating disorder and NoMAD questionnaires**

Table 1. Mean ratings and standard deviation of each item for the attitude questionnaire at baseline (Time 1), post-training (Time 2), and 3-month follow-up (Time 3).

| Attitude subscale | Early intervention for eating disorders… | Time | *n* | Mean (*SD*) | Strongly Disagree  (%, *n*) | Disagree  (%, *n*) | | Slightly Disagree  (%, *n*) | | Undecided  (%, *n*) | Slightly Agree  (%, *n*) | | Agree  (%, *n*) | | Strongly Agree  (%, *n*) |
| --- | --- | --- | --- | --- | --- | --- | --- | --- | --- | --- | --- | --- | --- | --- | --- |
| Positive | will improve patient outcomes in early-stage EDs | 1 | 186 | 6.46 (0.77) | 1% (1) | 0% (0) | | 1% (1) | | 2% (3) | 1% (2) | | 41% (77) | | 55% (102) |
|  |  | 2 | 152 | 6.54 (0.96) | 2% (3) | 0% (0) | | 0% (0) | | 1% (1) | 1% (2) | | 30% (45) | | 66% (101) |
|  |  | 3 | 114 | 6.57 (0.80) | 1% (1) | 0% (0) | | 0% (0) | | 2% (2) | 1% (1) | | 31% (35) | | 66% (75) |
|  | will reduce disruption to work, education, and social relationships of early-stage ED patients | 1 | 184 | 5.60 (1.53) | 3% (5) | 4% (8) | | 2% (4) | | 14% (25) | 5% (10) | | 41% (76) | | 30% (56) |
|  |  | 2 | 152 | 6.16 (1.08) | 0% (0) | 3% (5) | | 0% (0) | | 6% (9) | 0% (0) | | 49% (75) | | 41% (63) |
|  |  | 3 | 113 | 5.89 (1.33) | 1% (1) | 4% (4) | | 3% (3) | | 7% (8) | 8% (9) | | 40% (45) | | 38% (43) |
|  | will reduce the long-term economic costs of EDs | 1 | 184 | 5.97 (1.06) | 1% (1) | 1% (2) | | 0% (0) | | 8% (15) | 14% (26) | | 42% (77) | | 34% (63) |
|  |  | 2 | 152 | 6.08 (1.01) | 1% (1) | 1% (2) | | 0% (0) | | 6% (9) | 7% (10) | | 51% (77) | | 35% (53) |
|  |  | 3 | 113 | 6.09 (0.97) | 0% (0) | 1% (1) | | 0% (0) | | 9% (10) | 7% (8) | | 46% (52) | | 37% (42) |
|  | will improve the treatment uptake of early-stage ED patients | 1 | 184 | 5.83 (1.02) | 1% (2) | 0% (0) | | 0% (0) | | 11% (20) | 13% (23) | | 53% (98) | | 22% (41) |
|  |  | 2 | 151 | 6.28 (0.73) | 0% (0) | 0% (0) | | 0% (0) | | 4% (6) | 4% (6) | | 52% (78) | | 40% (61) |
|  |  | 3 | 114 | 6.11 (1.01) | 1% (1) | 1% (1) | | 1% (1) | | 4% (4) | 8% (9) | | 49% (56) | | 37% (42) |
|  | will reduce waiting times for early-stage ED patients | 1 | 184 | 5.99 (1.09) | 1% (1) | 2% (3) | | 1% (2) | | 7% (13) | 8% (14) | | 48% (89) | | 34% (62) |
|  |  | 2 | 151 | 6.03 (0.80) | 1% (1) | 1% (1) | | 0% (0) | | 2% (3) | 3% (5) | | 40% (61) | | 53% (80) |
|  |  | 3 | 114 | 6.25 (1.01) | 0% (0) | 3% (3) | | 0% (0) | | 4% (4) | 4% (5) | | 42% (48) | | 47% (54) |
|  | will reduce the burden on family, friends, or carers | 1 | 181 | 5.87 (0.95) | 0% (0) | 1% (1) | | 0% (0) | | 12% (22) | 11% (20) | | 53% (96) | | 24% (44) |
|  |  | 2 | 151 | 6.03 (0.80) | 0% (0) | 0% (0) | | 0% (0) | | 7% (10) | 11% (16) | | 56% (84) | | 27% (41) |
|  |  | 3 | 114 | 6.08 (0.92) | 0% (0) | 0% (0) | | 2% (2) | | 5% (6) | 12% (14) | | 45% (51) | | 36% (41) |
| Negative | will increase waiting times for other patients | 1 | 185 | 3.91 (1.46) | 5% (9) | 17% (31) | | 8% (14) | | 44% (82) | 9% (16) | | 15% (28) | | 3% (5) |
|  |  | 2 | 152 | 3.84 (1.49) | 4% (6) | 22% (33) | | 9% (14) | | 36% (54) | 15% (23) | | 11% (16) | | 4% (6) |
|  |  | 3 | 114 | 4.04 (1.65) | 6% (7) | 18% (20) | | 10% (11) | | 27% (31) | 18% (21) | | 15% (17) | | 6% (7) |
|  | will result in the overtreatment of short-lived or mild eating, weight, and shape concerns | 1 | 184 | 2.93 (1.40) | 10% (19) | 41% (75) | | 14% (26) | | 23% (42) | 6% (11) | | 4% (8) | | 2% (3) |
|  |  | 2 | 152 | 2.57 (1.34) | 19% (29) | 45% (69) | | 9% (14) | | 16% (25) | 6% (9) | | 4% (6) | | 0% (0) |
|  |  | 3 | 114 | 2.64 (1.43) | 19% (22) | 42% (48) | | 11% (13) | | 16% (18) | 7% (8) | | 3% (3) | | 2% (2) |
|  | will increase the demand on teams | 1 | 185 | 4.49 (1.45) | 1% (2) | 12% (22) | | 8% (14) | | 30% (56) | 23% (42) | | 19% (35) | | 8% (14) |
|  |  | 2 | 151 | 4.30 (1.47) | 3% (4) | 13% (20) | | 9% (14) | | 29% (44) | 23% (34) | | 19% (28) | | 5% (7) |
|  |  | 3 | 113 | 4.47 (1.58) | 3% (3) | 16% (18) | | 4% (5) | | 22% (25) | 26% (29) | | 22% (25) | | 7% (8) |
|  | will divert valuable resources away from those with more severe/enduring forms of the illness | 1 | 184 | 3.33 (1.37) | 5% (10) | 32% (58) | | 14% (26) | | 29% (54) | 14% (25) | | 5% (9) | | 1% (2) |
|  |  | 2 | 152 | 3.26 (1.47) | 7% (11) | 33% (50) | | 18% (27) | | 22% (34) | 9% (14) | | 10% (15) | | 1% (1) |
|  |  | 3 | 113 | 3.21 (1.67) | 13% (15) | 33% (37) | | 14% (16) | | 13% (15) | 15% (17) | | 9% (10) | | 3% (3) |
|  | It is best to adopt a ‘watch and wait’ approach for people with milder ED symptoms | 1 | 182 | 2.43 (1.22) | 21% (38) | 46% (83) | | 11% (20) | | 15% (28) | 7% (12) | | 0% (0) | | 1% (1) |
|  |  | 2 | 151 | 2.27 (1.24) | 26% (39) | 48% (73) | | 10% (15) | | 7% (11) | 6% (9) | | 3% (4) | | 0% (0) |
|  |  | 3 | 114 | 2.31 (1.36) | 28% (32) | 44% (50) | | 11% (13) | | 9% (10) | 4% (4) | | 3% (3) | | 2% (2) |
| Knowledge and Skills | I am aware of the rationale, principles, and ways of delivering early intervention for EDs | 1 | 183 | 5.52 (1.21) | 1% (1) | 4% (8) | | 2% (4) | | 7% (13) | 22% (40) | | 49% (90) | | 15% (27) |
|  |  | 2 | 151 | 6.36 (0.59) | 0% (0) | 0% (0) | | 0% (0) | | 1% (1) | 4% (6) | | 54% (81) | | 42% (63) |
|  |  | 3 | 114 | 6.19 (0.92) | 1% (1) | 0% (0) | | 2% (2) | | 2% (2) | 5% (6) | | 53% (60) | | 38% (43) |
|  | I have the clinical skills to deliver interventions that are tailored to the illness and developmental stage of early ED patients | 1 | 182 | 5.31 (1.32) | 3 (2%) | 5% (9) | | 1% (1) | | 15% (27) | 22% (40) | | 43% (79) | | 13% (23) |
|  |  | 2 | 150 | 5.87 (1.09) | 1% (2) | 1% (2) | | 1% (1) | | 5% (7) | 15% (22) | | 52% (78) | | 25% (38) |
|  |  | 3 | 114 | 5.94 (1.08) | 2% (2) | 0% (0) | | 1% (1) | | 4% (5) | 16% (18) | | 47% (54) | | 30% (34) |
| Importance of Early Intervention for EDs | How important do you consider early intervention for the following disorders? |  |  |  | Not Important  (%, *n*) | | Slightly Important  (%, *n*) | | Moderately Important  (%, *n*) | | | Important  (%, *n*) | | Absolutely Essential  (%, *n*) | |
|  | Anorexia Nervosa | 1 | 180 | 4.79 (0.45) | 0% (0) | | 1% (1) | | 0% (0) | | | 19% (34) | | 81% (145) | |
|  |  | 2 | 146 | 4.79 (0.41) | 0% (0) | | 0% (0) | | 0% (0) | | | 20% (30) | | 80% (116) | |
|  |  | 3 | 112 | 4.86 (0.38) | 0% (0) | | 0% (0) | | 1% (1) | | | 13% (14) | | 87% (97) | |
|  | Other Eating Disorders | 1 | 181 | 4.52 (0.61) | 0% (0) | | 1% (2) | | 3% (5) | | | 39% (71) | | 57% (103) | |
|  |  | 2 | 148 | 4.59 (0.58) | 0% (0) | | 1% (2) | | 1% (1) | | | 35% (52) | | 63% (93) | |
|  |  | 3 | 114 | 4.60 (0.54) | 0% (0) | | 0% (0) | | 3% (3) | | | 35% (40) | | 62% (71) | |

*Notes.* ED = Eating Disorders.

Table 2. Mean ratings and standard deviation of each item for the NoMAD questionnaire at baseline (Time 1), post-training (Time 2), and 3-month follow-up (Time 3).

| NPT Construct | NoMAD Item | Time | *n* | Mean (*SD*) | Strongly Disagree  (%, *n*) | Disagree  (%, *n*) | Neither Agree nor Disagree  (%, *n*) | Agree  (%, *n*) | Strongly Agree  (%, *n*) | Not Applicable to Role, Stage or Intervention  (%, *n*) |
| --- | --- | --- | --- | --- | --- | --- | --- | --- | --- | --- |
| Coherence | I can see how FREED differs from usual ways of working | 1 | 185 | 3.99 (0.70) | 0% (0) | 4% (7) | 11% (20) | 57% (106) | 18% (33) | 10% (19) |
|  |  | 2 | 154 | 4.16 (0.71) | 0% (0) | 3% (4) | 10% (15) | 55% (84) | 31% (47) | 3%(4) |
|  |  | 3 | 115 | 4.09 (0.82) | 3% (3) | 3% (3) | 5% (6) | 60% (69) | 27% (31) | 3% (3) |
|  | Staff in this organisation have a shared understanding of the purpose of FREED | 1 | 185 | 3.63 (0.84) | 1% (2) | 7% (12) | 27% (50) | 43% (80) | 11% (20) | 11% (21) |
|  |  | 2 | 152 | 4.00 (0.82) | 0% (0) | 7% (11) | 11% (16) | 53% (81) | 25% (38) | 4% (6) |
|  |  | 3 | 115 | 4.07 (0.79) | 0% (0) | 5% (6) | 11% (13) | 51% (59) | 29% (33) | 3% (4) |
|  | I understand how FREED affects the nature of my work | 1 | 185 | 3.81 (0.78) | 0% (0) | 6% (11) | 16% (30) | 48% (89) | 12% (23) | 17% (32) |
|  |  | 2 | 154 | 4.19 (0.62) | 0% (0) | 1% (1) | 8% (13) | 57% (87) | 28% (43) | 7% (9) |
|  |  | 3 | 115 | 4.20 (0.67) | 0% (0) | 1% (1) | 10% (12) | 10% (12) | 49% (56) | 10% (11) |
|  | I can see the potential value of FREED for my work | 1 | 185 | 4.53 (0.57) | 0% (0) | 0% (0) | 3% (6) | 37% (68) | 52% (96) | 8% (15) |
|  |  | 2 | 154 | 4.57 (0.55) | 0% (0) | 0% (0) | 3% (4) | 36% (56) | 58% (90) | 3% (4) |
|  |  | 3 | 114 | 4.57 (0.52) | 0% (0) | 0% (0) | 1% (1) | 40% (46) | 56% (64) | 3% (3) |
| Cognitive Participation | There are key people who drive FREED forward and get others involved | 1 | 184 | 4.26 (0.65) | 0% (0) | 0% (0) | 10% (18) | 43% (79) | 32% (59) | 15% (28) |
|  |  | 2 | 154 | 4.39 (0.65) | 0% (0) | 1% (2) | 5% (7) | 43% (66) | 43% (66) | 8% (13) |
|  |  | 3 | 114 | 4.44 (0.63) | 0% (0) | 2% (2) | 2% (2) | 45% (51) | 47% (53) | 5% (6) |
|  | I believe that participating in FREED is a legitimate part of my role | 1 | 185 | 4.33 (0.66) | 0% (0) | 1% (1) | 8% (14) | 42% (78) | 37% (69) | 12% (23) |
|  |  | 2 | 154 | 4.39 (0.63) | 0% (0) | 1% (1) | 5% (8) | 43% (66) | 42% (64) | 10% (15) |
|  |  | 3 | 115 | 4.49 (0.59) | 0% (0) | 0% (0) | 4% (5) | 38% (44) | 49% (56) | 9% (10) |
|  | I’m open to working with colleagues in new ways to implement FREED | 1 | 185 | 4.63 (0.50) | 0% (0) | 0% (0) | 1% (1) | 34% (63) | 61% (112) | 5% (9) |
|  |  | 2 | 154 | 4.66 (0.52) | 0% (0) | 0% (0) | 2% (3) | 29% (44) | 66% (101) | 4% (6) |
|  |  | 3 | 115 | 4.69 (0.47) | 0% (0) | 0% (0) | 0% (0) | 30% (35) | 67% (77) | 3% (3) |
|  | I will continue to support FREED | 1 | 185 | 4.54 (0.56) | 0% (0) | 0% (0) | 3% (5) | 37% (69) | 52% (96) | 8% (15) |
|  |  | 2 | 154 | 4.65 (0.51) | 0% (0) | 0% (0) | 1% (2) | 31% (48) | 64% (99) | 3% (5) |
|  |  | 3 | 115 | 4.66 (0.49) | 0% (0) | 0% (0) | 1% (1) | 31% (36) | 65% (75) | 3% (3) |
| Collective Action | I can integrate FREED into my existing work | 1 | 185 | 4.28 (0.68) | 0% (0) | 1% (1) | 9% (17) | 42% (77) | 35% (64) | 14% (26) |
|  |  | 2 | 154 | 4.35 (0.67) | 0% (0) | 1% (1) | 8% (12) | 41% (63) | 41% (63) | 10% (15) |
|  |  | 3 | 115 | 4.30 (0.80) | 1% (1) | 1% (1) | 10% (12) | 33% (38) | 41% (47) | 14% (16) |
|  | FREED disrupts working relationships | 1 | 185 | 1.90 (0.79) | 28% (51) | 36% (66) | 17% (32) | 1% (2) | 0% (0) | 18% (34) |
|  |  | 2 | 153 | 1.81 (0.80) | 32% (49) | 41% (63) | 11% (16) | 1% (1) | 1% (2) | 14% (22) |
|  |  | 3 | 115 | 1.63 (0.72) | 41% (47) | 32% (37) | 9% (10) | 1% (1) | 0% (0) | 17% (20) |
|  | I have confidence in other people’s ability to implement FREED | 1 | 183 | 4.01 (0.74) | 0% (0) | 3% (5) | 16% (29) | 49% (90) | 22% (41) | 10% (18) |
|  |  | 2 | 151 | 4.14 (0.69) | 1% (1) | 1% (1) | 10% (15) | 54% (82) | 27% (40) | 8% (12) |
|  |  | 3 | 114 | 4.13 (0.81) | 2% (2) | 1% (1) | 12% (14) | 48% (55) | 32% (36) | 5% (6) |
|  | Work is assigned to those with skills appropriate to using FREED | 1 | 185 | 3.80 (0.79) | 2% (3) | 1% (1) | 21% (39) | 40% (73) | 12% (22) | 25% (47) |
|  |  | 2 | 152 | 3.87 (0.83) | 2% (3) | 2% (3) | 17% (26) | 47% (72) | 16% (24) | 16% (24) |
|  |  | 3 | 113 | 4.05 (0.84) | 0% (0) | 4% (5) | 13% (15) | 38% (43) | 27% (30) | 18% (20) |
|  | Sufficient training is provided to enable staff to implement FREED | 1 | 186 | 3.80 (0.76) | 0% (0) | 3% (6) | 22% (41) | 40% (75) | 13% (24) | 22% (40) |
|  |  | 2 | 153 | 4.21 (0.62) | 0% (0) | 1% (1) | 8% (12) | 56% (86) | 29% (44) | 7% (10) |
|  |  | 3 | 115 | 4.09 (0.85) | 1% (1) | 4% (4) | 13% (15) | 44% (50) | 30% (35) | 9% (10) |
|  | Sufficient resources are available to support FREED | 1 | 185 | 3.41 (1.13) | 5% (10) | 8% (15) | 27% (49) | 21% (38) | 15% (27) | 25% (46) |
|  |  | 2 | 152 | 4.00 (1.01) | 3% (4) | 7% (10) | 13% (20) | 37% (56) | 34% (52) | 7% (10) |
|  |  | 3 | 115 | 3.76 (1.21) | 7% (8) | 10% (11) | 11% (13) | 37% (43) | 29% (33) | 6% (7) |
|  | Management adequately support FREED | 1 | 185 | 4.03 (0.85) | 1% (2) | 1% (2) | 18% (34) | 35% (65) | 27% (49) | 18% (33) |
|  |  | 2 | 153 | 4.09 (0.81) | 1% (2) | 2% (3) | 11% (17) | 46% (70) | 27% (41) | 13% (20) |
|  |  | 3 | 115 | 4.17 (0.90) | 2% (2) | 3% (3) | 12% (14) | 37% (42) | 38% (44) | 9% (10) |
| Reflexive Monitoring | I am aware of reports about the effects of FREED | 1 | 184 | 3.92 (0.85) | 1% (1) | 8% (14) | 11% (20) | 52% (96) | 20% (37) | 9% (16) |
|  |  | 2 | 153 | 4.23 (0.68) | 0% (0) | 2% (3) | 8% (12) | 53% (81) | 35% (53) | 3% (4) |
|  |  | 3 | 115 | 4.21 (0.72) | 1% (1) | 1% (1) | 10% (11) | 54% (62) | 35% (40) | 0% (0) |
|  | The staff agree that FREED is worthwhile | 1 | 184 | 4.10 (0.73) | 0% (0) | 2% (4) | 13% (24) | 47% (87) | 26% (48) | 11% (21) |
|  |  | 2 | 152 | 4.26 (0.62) | 0% (0) | 1% (2) | 5% (8) | 56% (85) | 33% (50) | 5% (7) |
|  |  | 3 | 114 | 4.41 (0.62) | 0% (0) | 1% (1) | 4% (5) | 46% (52) | 47% (53) | 3% (3) |
|  | I value the effects that FREED has on my work | 1 | 183 | 4.10 (0.76) | 0% (0) | 0% (0) | 13% (24) | 22% (41) | 19% (34) | 46% (84) |
|  |  | 2 | 153 | 4.17 (0.77) | 1% (1) | 0% (0) | 11% (16) | 31% (47) | 24% (36) | 35% (53) |
|  |  | 3 | 115 | 4.17 (0.83) | 0% (0) | 2% (2) | 17% (19) | 29% (33) | 34% (39) | 19% (22) |
|  | Feedback about FREED can be used to improve it in the future | 1 | 186 | 4.43 (0.54) | 0% (0) | 0% (0) | 2% (4) | 48% (89) | 41% (77) | 9% (16) |
|  |  | 2 | 153 | 4.53 (0.54) | 0% (0) | 0% (0) | 2% (3) | 41% (62) | 52% (80) | 5% (8) |
|  |  | 3 | 114 | 4.56 (0.50) | 0% (0) | 0% (0) | 0% (0) | 43% (49) | 55% (63) | 2% (2) |
|  | I can modify how I work with FREED | 1 | 185 | 3.99 (0.68) | 0% (0) | 0% (0) | 17% (31) | 40% (74) | 16% (30) | 27% (50) |
|  |  | 2 | 153 | 4.22 (0.68) | 1% (1) | 0% (0) | 9% (13) | 49% (75) | 30% (46) | 12% (18) |
|  |  | 3 | 115 | 4.23 (0.69) | 0% (0) | 2% (2) | 7% (8) | 45% (51) | 30% (34) | 17% (19) |

Note. FREED = First Episode Rapid Early Intervention for Eating Disorders. All items rated on a 5-point Likert scale ranging from ‘Strongly Disagree’ (1) to ‘Strongly Agree’.
